# Supplementary material for: Magnetoresistive-coupled transistor using the Weyl semimetal NbP
Source: Nat Commun. 2024 Jan 24;15:710. doi: 10.1038/s41467-024-44961-5 (PMC11258312; doi:10.1038/s41467-024-44961-5)
Supplement: Supplementary file 1 — Supplementary Information [file 41467_2024_44961_MOESM1_ESM.pdf]

Supplementary information for

Magnetoresistive-coupled transistor using the Weyl semimetal NbP

Lorenzo Rocchino[1]*, Federico Balduini[1], Heinz Schmid[1], Alan Molinari[1], Mathieu Luisier[2], Vicky Süß[3], Claudia Felser[3], Bernd Gotsmann[1], Cezar B. Zota[1]

Affiliations:

[1]IBM Research Europe – Zürich, Saümerstrasse 4, 8803 Rüschlikon, Switzerland

[2]ETH Zurich, Rämistrasse 101, 8092 Zürich, Switzerland

[3]Max Planck Institute for Chemical Physics of Solids, Nöthnitzer Straße 40, 01187 Dresden, Germany

*Corresponding author. Email: lorenzo.rocchino@zurich.ibm.com

Derivation of the Transconductance Equation

Eq.1 in the main text is analytically derived by expressing the current flowing in the WSM I_{WSM} as a function of the gate voltage V_G:

$$g_m \overset{\text{def}}{=} \frac{\partial I_{out}}{\partial V_{in}} = \frac{\partial I_{WSM}}{\partial V_G} = \frac{\partial}{\partial V_G}\left[\frac{V_{WSM}}{R_{WSM}(B)}\right]$$

$$R_{WSM}(B) = \rho_{WSM}^0 \frac{l_{WSM}}{A_{WSM}}[1 + MR(B)] = K_{WSM}[1 + MR(B)]$$

Here, g_m is the transconductance, V_{WSM} is the voltage applied to the NbP crystallite, R_{WSM} is the NbP resistance and ρ_{WSM}^0 the zero-field resistivity. A_{WSM} and l_{WSM} are the cross-section area and the length of the active region of the NbP crystallite, respectively. Assuming that the magnetoresistance (MR) varies linearly with the magnetic field strength B, it holds:

$$MR(B) \cong \alpha B(V_G) = \alpha \frac{\mu_0 I_{BS}}{2\pi Z_G A_G} V_G = K_G V_G$$

Here, α is the slope of the MR, μ_0 is the magnetic permittivity of vacuum, I_{BS} is the result of the Biot-Savart integral over the volume of the NbP crystallite, Z_G is the gate impedence and A_G the gate cross-section area. The final expression of g_m can be obtained by computing the derivative:

$$g_m = \frac{V_{WSM}}{K_{WSM}} \frac{\partial}{\partial V_G}\left(\frac{1}{1+K_G V_G}\right) = -\frac{V_{WSM}}{K_{WSM}} \frac{K_G}{(1+K_G V_G)^2}$$

Relationship between Oscillation Frequency and Effective Mass

The quantities mentioned in the main text are evaluated from the data extracted from quantum oscillations analysis according to the following relationships:

$$A = \frac{2\pi eF}{\hbar}, \quad k_F = \sqrt{\frac{A}{\pi}}, \quad n = \frac{{k_F}^3}{3\pi^2}, \quad \tau = \frac{m_{eff}}{e^2 n\rho}, \quad \mu = \frac{e\tau}{m_{eff}}, \quad \lambda = \frac{\hbar k_F}{m_{eff}}$$

Here, A is the area of the cross-section of the Fermi surface, e is the electron charge, $\hbar$ is the reduced Plank's constant, k_F is the Fermi momentum, n is the carrier density, τ is the quantum relaxation time, m_{eff} is the effective mass, ρ is the electrical resistivity, μ is the carrier mobility and λ is the mean free path.

Frequency Behavior and Signal Transfer

In the transconductance equation of the, V_G is a function of the gate impedance, as the device is current-driven and the gate is in its superconductive state (therefore, its intrinsic resistance is equal to zero). In particular it holds that:

$$Z_G = R_G + i\omega L_K$$

Where Z_G is the total gate impedance, R_G is the real component of the impedance (which will be given by the contacts), i is the imaginary unit, ω is the frequency and finally L_K is the kinetic inductance. Its value can be estimated in the framework of the Ginzburg-Landau theory as:

$$L_K = \frac{m_e}{2n_s e^2}\frac{l_G}{A_G}$$

Here, m_e is the electron mass, n_s is the density of Cooper pairs, l_G is the length of the gate and A_G its cross-sectional area.

The overall device performance and frequency behavior depends on the quality of the signal transfer from the gate to the WSM (i.e., on Z_G). In particular, at low frequency, the real component of the impedance will be dominant (hence, the contacts resistance), while at higher frequency the reactive part will play a major role. The impact of Z_G becomes even more relevant if we look at gain metrics (e.g., $A_I = Z_G \cdot g_m$, with A_I being the current gain). In fact. Z_G is in principle zero in DC, therefore the current gain is not well-defined in the ideal case but depends on the real R_G. On a similar note, the voltage gain, A_V, can be evaluated as: $A_V = R_{WSM} \cdot g_m$. The developed device has a voltage gain below the unit due to the small resistance

of the WSM. A more detailed analysis of gain metrics and frequency behavior has been discussed in a previous work[1].

[1] Toniato, A., Gotsmann, B., Lind, E. & Zota, C. B. Weyl Semi-Metal-Based High-Frequency Amplifiers. in 2019 IEEE International Electron Devices Meeting (IEDM) 9.4.1-9.4.4 (2019). doi:10.1109/IEDM19573.2019.8993575.

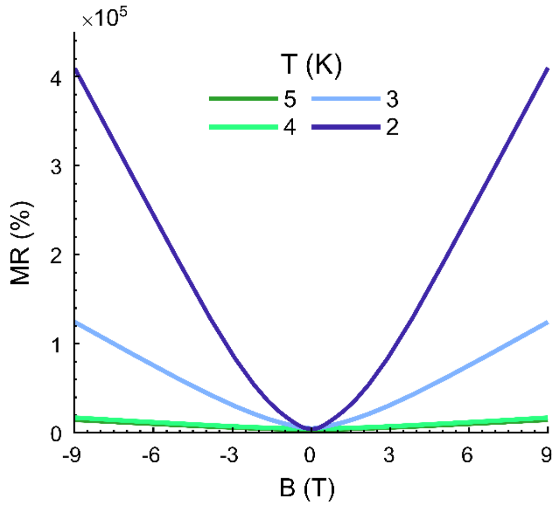

Fig. S1 | MR in the superconductive region. The apparent increase of the MR is due to a lowered zero-field resistivity and not to an intrinsic magnetic response.

a

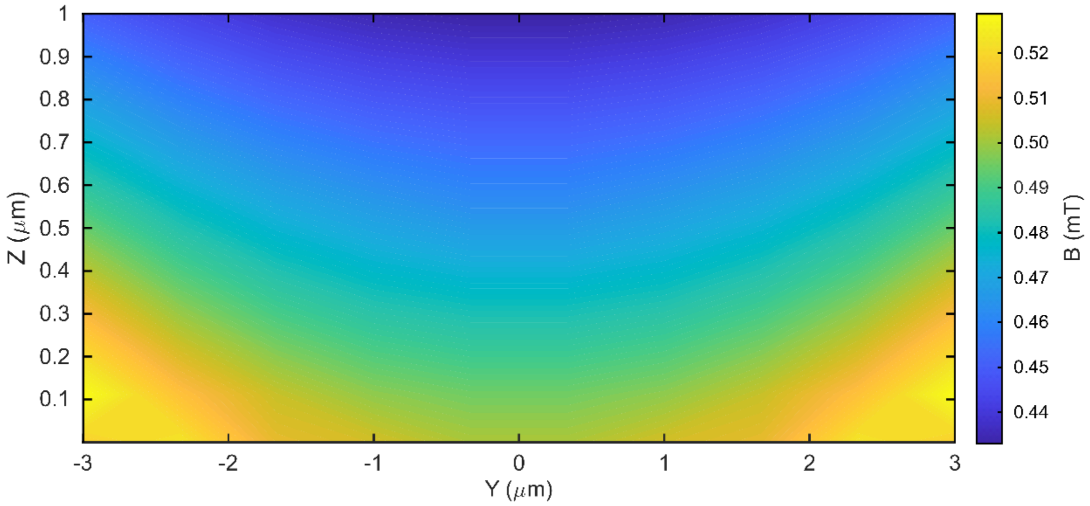

b

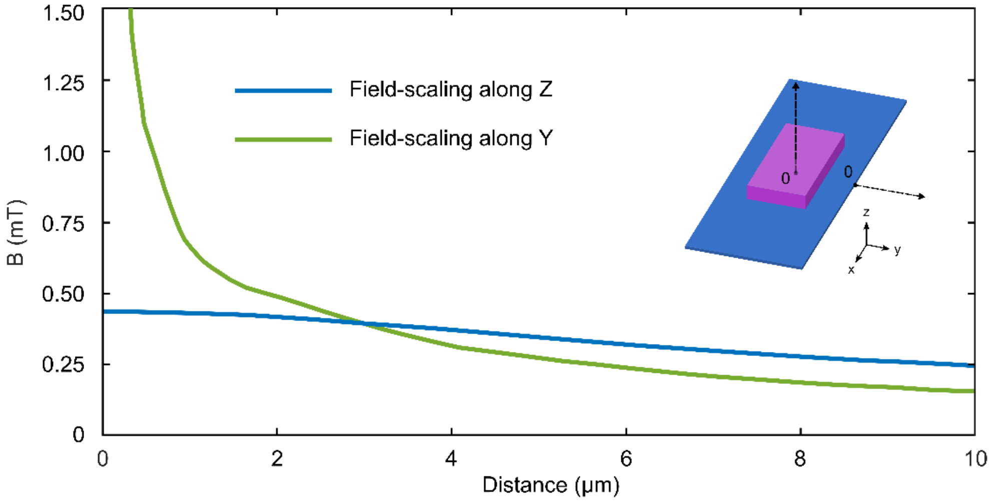

Fig. S2 | Numerical simulation of the gate-generated field strength in a transverse cross-section of the NbP channel. a, The average magnetic field strength is equal to 0.48 mT. The computed field strength is generated by a gate current of 13.6 mA. The uniformity degree of the field strength (By/Bz) is 92.73%. **b,** In plane (y direction) and out-of-plane (z direction) field scaling as a function of the distance from the gate. Outside of the device active area, the field vanishes more rapidly in the xy-plane than in the z direction. Vertical scaling is mitigated by the aspect ratio of the gate which favors the width over the thickness.

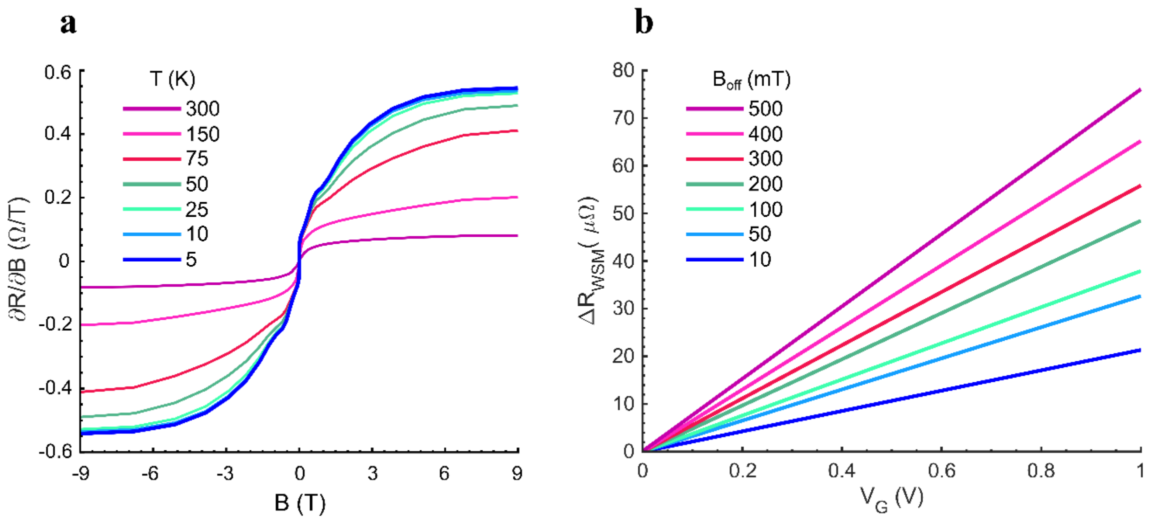

Fig. S3 | First derivative of the transverse magnetoresistance and resistance modulation for different field offsets. a, A sharp parabolic to linear transition is clearly visible at room temperature and it occurs around 1 T. Upon decreasing T, the transition between the two regimes is much broader. A steep increase in the modulation capability is observed at all temperatures in the first hundreds mT. **b,** Interpolated data of the resistance modulation as a function of the applied gate voltage. A higher field offset lifts the operation point towards steeper section of the MR curve.

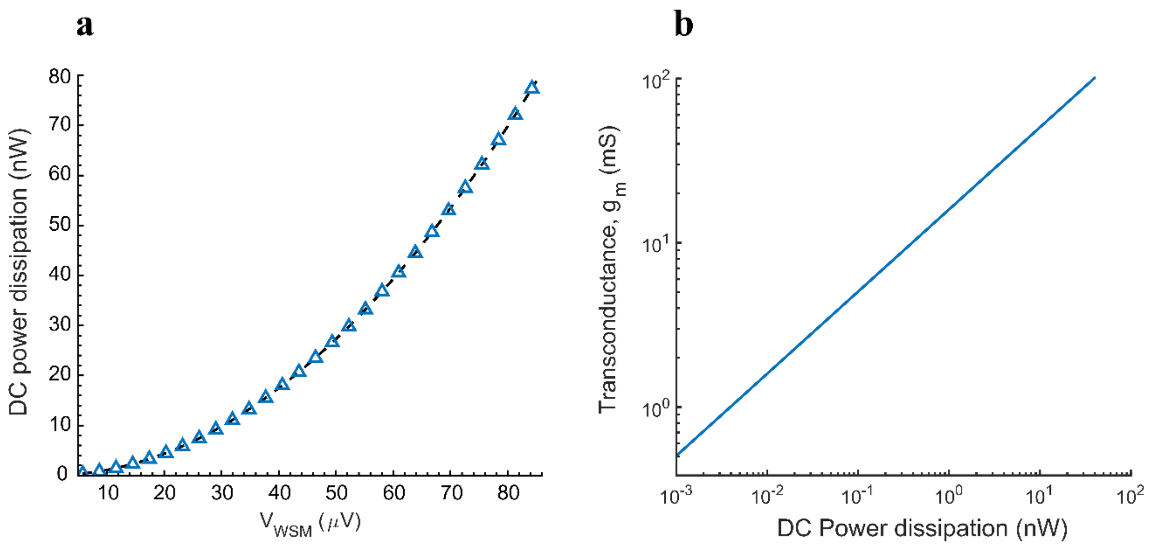

Fig. S4 | DC power dissipation. a, Power dissipation as a function of the applied WSM voltage, taken at T = 5 K with an external field offset of 500 mT. **b,** Logarithmic plot of the transconductance as a function of the WSM power.

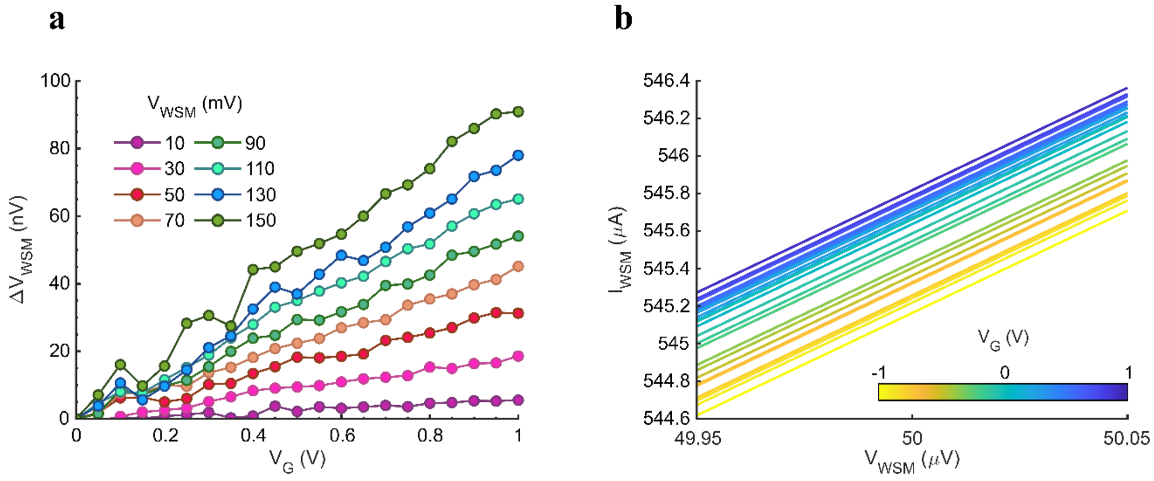

Fig. S5 | Transfer and output characteristics. a, Modulation of the WSM voltage as a function of the gate voltage for different nominal biases. From this dataset it is possible to extract the transconductance reported in Fig. 4b. **b,** Modulation of the WSM current for increasing gate voltage (-1 V to 1 V) at the bias point.

	Length (µm)	*Width (µm)*	*Thickness (nm)*
NbN	/	12	200
NbP	9	6	1000
Pt contacts	n.a.	n.a.	1000[2]
SiO_2	/	/	20

Tab. S1 | Dimensions of the active area of the Weyl transistor.

[2] Estimation based on the known deposition ratio of Pt. The actual thickness was not directly measured.

	Measured frequency (T)	*Predicted frequency (T)*	*Fermi momentum (Å^{-1})*	*Carrier density (cm^{-3})*	*Mean free path (µm)*	*Effective mass (m_e)*
E_1	18.6	13	0.036	$4.7 \cdot 10^{17}$	99.3	0.14
E_1	30.1	34	0.030	$8.6 \cdot 10^{17}$	61.3	0.09
H_1	42.7	41	0.024	$1.8 \cdot 10^{18}$	43.1	0.11

Tab. S2 | Physical quantities extracted from quantum oscillations analysis.
